# Prevalence and Risk Factors for Acute Kidney Injury Among Adults Undergoing Cardiac Interventions in King Abdulaziz University Hospital: A Retrospective Review

**DOI:** 10.7759/cureus.23387

**Published:** 2022-03-22

**Authors:** Khalid G Almramhi, Moussa A Alkhateeb, Omar A Alsulami, Saeed A Alhudaifi, Hamza Alamoudi, Rana A Nabalawi

**Affiliations:** 1 Internal Medicine, King Abdulaziz University Faculty of Medicine, Jeddah, SAU; 2 Nephrology, King Abdulaziz University Faculty of Medicine, Jeddah, SAU

**Keywords:** comorbidities, cardiac interventions, acute kidney injury, risk factors, prevalence

## Abstract

Background

Acute kidney injury (AKI) is a syndrome that has been receiving considerable attention as a common risk in cardiac surgeries, which has consequences for short- and long-term survival implications, even for those who do not progress to renal failure. There have been limited studies in the Middle East, and specifically in the Kingdom of Saudi Arabia (KSA). Therefore, our study aimed to identify the prevalence of and risk factors for AKIs following adult cardiac interventions during 2010-2020 at the King Abdulaziz University Hospital (KAUH), Jeddah, Saudi Arabia.

Methods

Setting and Design: A retrospective medical record review was conducted among all the adult patients who underwent cardiac interventions and developed AKIs between 2010 and 2020. Google forms were used to extract the data from the hospital records. About statistical analysis binary logistic regression analyses, relative risks (RRs), and confidence intervals (CI) were used to determine the associations among the variables.

Results

After applying the inclusion and exclusion criteria, 564 patients were included. Their baseline demographic, clinical, biological, and operative characteristics were analyzed. AKIs developed in 110 (19.5%) patients and patients with diabetes were more likely to develop AKIs (*P *< 0.012, RR = 2.280, CI = 1.198-4.339). Hypertension showed a strong effect in the development of AKIs (*P *< 0.004, RR = 2.865, CI = 1.391-5.900). Moreover, patients who suffered from chronic heart failure were more prone to the development of AKIs (*P *< 0.008, RR = 4.189, CI =1.452-12.087). Furthermore, anemia with significant *P-*values (<0.002), and CIs of 1.509-6.822, indicated that these patients were more likely to develop AKIs (3.209 times).

Conclusion

We demonstrated that AKIs are frequent complications in adults after cardiac interventions and were associated with poor outcomes. Risk factors for the development of AKIs were identified to be diabetes mellitus, hypertension, chronic heart failure, and anemia. Further investigation of this cohort is necessary to better understand the problem of kidney injuries.

## Introduction

Acute kidney injury (AKI) is a syndrome that has been receiving considerable attention as a common risk in cardiac surgeries, which has consequences for short- and long-term survival implications, even for those who do not progress to renal failure [1]. Additionally, the risk for mortality increases exponentially among patients who develop postoperative AKIs with mortality rates in excess of 60% [2-6]. AKI is characterized by the rapid loss of the kidney’s excretory function, and it’s diagnosed typically by the accumulation of end products of nitrogen metabolism (urea and creatinine) or decreased urine output, or both [7]. Increases in serum creatinine are associated with an increased risk of mortality, even if they are minor increases [8-15]. Annually, over two million cardiac interventions occur among adult patients [16]. The most common complication being AKI, which increases the incidence of morbidity and mortality, whether due to coronary angiographies, which are considered to be the third leading cause of hospital-acquired AKIs or coronary pulmonary bypass surgeries (CPBs) of which 20-30% cause AKIs [17-19].

Nevertheless, AKI is reported to occur in up to 30% of patients who have undergone heart surgery, and 1% to 2% of those with heart surgery require renal replacement treatment (RRT) [19-21]. However, early interventions at the individual, population, regional, and in-hospital levels may minimize the occurrence of AKIs. Moreover, the prediction and prevention of AKIs are significant healthcare priorities because once AKIs have developed, the therapeutic options are minimized [22,23]. A previous study that was conducted in Washington, USA, with a sample size of 985,737 consecutive patients who underwent percutaneous coronary interventions (PCIs), shows that AKIs developed in 69,658 (7.1%) of the patients with 3,005 (0.3%) of them requiring new dialysis [23]. Furthermore, another study showed that AKIs occurred in 328 (31.1%) of 1,056 consecutive patients undergoing cardiac surgeries at Renji Hospital, Shanghai, China [24]. And in another study from April 2008 to March 2009, which included 433 adult patients who underwent heart surgeries, 221 (49.9%) suffered postoperative AKIs [25]. Moreover, recent epidemiologic studies identified a wide range of causes and risk factors that have been linked to acute kidney injuries [26-28]. And that were the strongest predictors of cardiac surgery-associated acute kidney injury (CSA-AKI), such as old age, preoperative hypertension, preoperative anemia, left ventricular ejection fraction (LVEF) <45%, CPB times ≥110 minutes, transfused red blood cells (RBCs), postoperative hypotension, central venous pressure (CVP) <6 cmH_2_O, mechanical ventilation ≥9 hours, use of angiotensin converting enzyme inhibitors (ACEIs)/angiotensin-receptor blockers (ARBs), postoperative CVP <6 cmH_2_O, and hypotension [29].

Nonetheless, there have been limited studies in the Middle East, and specifically in the Kingdom of Saudi Arabia (KSA). Therefore, our study aimed to identify the prevalence and risk factors of AKIs following adult cardiac interventions during 2010-2020 at the King Abdulaziz University Hospital (KAUH), Jeddah, Saudi Arabia.

## Materials and methods

Setting and design

This 10-year retrospective record review was conducted during May-June 2021 after receiving approval from the Research Ethical Committee at the College of Medicine, KAUH, Jeddah, KSA (Reference: 228-21). KAUH is a government tertiary healthcare center that serves all strata of society. The focus of our study was to first to identify the most significant risk factors that led to AKIs after cardiac interventions and then to determine its prevalence.

Inclusion and exclusion criteria

We included all the adult (>18 years) patients who underwent cardiac interventions and developed AKIs during the period of 2010-2020 at KAUH.

Method and material

The data were collected manually from our hospital information system, Phoenix (Al-Anaiah International Co. Ltd., Jeddah, Saudi Arabia), using Google Forms. After excluding patients with end-stage renal disease on regular dialysis, we extracted the following variables from 564 patients: medical record number, nationality, gender, age at the time of surgery, developed AKI after cardiac intervention (Yes-No), any associated comorbidities (diabetes mellitus (DM), hypertension (HTN), ischemic heart disease (IHD), chronic heart failure (CHF); malignancies: chronic kidney disease (CKD), cardiovascular disease (CVD), thyroid disease, autoimmune disease, peripheral vascular disease (PVD), chronic liver disease, liver cirrhosis, respiratory disease, anemia, hyperlipidemia, obesity, smoking), type of cardiac intervention (coronary artery bypass graft (CABG) or percutaneous transluminal coronary angioplasty (PTCA)), priority of surgery (elective or emergency), date and time of surgery, length of hospitalization, surgery duration, LVEF, patient BMI, and preoperative and postoperative lab assessment (preoperative and postoperative creatinine, preoperative blood urea nitrogen (BUN), preoperative Hb, preoperative hematocrit (HCT)).

Statistical analysis used

Microsoft Excel v16.0 was used to organize the data, which were then analyzed statistically using IBM SPSS Statistics for Windows (Version 21.0; IBM Corp., Armonk, NY, USA). For the descriptive statistics, the continuous variables were summarized using means and associated standard deviations (SDs), while the categorical variables were presented using numbers and associated frequencies. The differences in the clinical characteristics and outcomes between patients with or without AKIs were tested using the student’s t-test for the continuous variables and a chi-square test or Fisher’s exact test for the categorical variables. Preoperative and intraoperative risk factors for kidney injuries were identified using univariable and multivariable logistic regressions, and the results were presented as relative risks (RR) with 95% CIs. P-values <0.05 were deemed significant.

## Results

Patient population and sample attrition

After applying the inclusion and exclusion criteria, 74 patients (pediatric patients, patients in end-stage renal disease, and on dialysis) were excluded and 564 patients were included. Their baseline demographic, clinical, biological, and operative characteristics were analyzed in this retrospective cohort study. We targeted those who developed AKIs after cardiac interventions in KAUH in Jeddah, Saudi Arabia, between 2010 to 2020.

Demographic and descriptive characteristic of patients

In the overall sample, the mean age was 61.6± 9.9 years, males were predominant 481 (85.3%) comparatively. Female patients accounted for 83 (14.7%) of the total number of patients, with 34.9% of them having AKIs (Table 1). Non-Saudi patients were the most abundant in terms of nationality 473 (84.5%) as well having the most significant number of AKI patients, 87 (80.6%). The cardiac surgeries in this sample were CABG in 407 (72.2%), and PTCA in 157 (27.8%). Moreover, 492(87.2%) of the surgeries were elective. The mean and standard deviation of the length of hospitalization stay was 6.1 ± 6.3 days, and the patients’ BMIs was 27.43±43.43 (Table 1).

**Table 1:** Demographic and Descriptive Characteristic of Patients CABG: coronary artery bypass graft surgery; PTCA: percutaneous transluminal coronary angioplasty; LVEF: left ventricular ejection fraction; LOH: length of hospitality; BMI: body mass index

Variable	Total Number: N = 564 (100%)	Develop AKI: N = 110 (19.5%)	Non-develop AKI: N = 454 (80.5%)
Gender(1): Male	481 (85.3%)	81 (73.6%)	400 (88.1%)
gender(2): Female	83 (14.7%)	29 (26.4%)	54 (11.9%)
Nationality(1): Saudi	87 (15.5%)	21 (19.4%)	66 (14.6%)
Nationality(2): Non-Saudi	473 (84.5%)	87 (80.6%)	386 (85.4%)
Priority of surgery(1): Elective	492 (87.2%)	81 (73.6%)	411 (90.5%)
Priority of surgery (2): emergency	72 (12.8%)	29 (26.4%)	43 (9.5%)
Type of surgery(1): CABG	407 (72.2%)	100 (90.9%)	307 (67.6%)
Type of surgery(2): PTCA	157 (27.8%)	10 (9.1%)	149 (32.4%)
Descriptive	All (Mean±SD)	Develop AKI (Mean±SD)	Non-develop AKI (Mean±SD)
Age (years)	61.64±9.97	63.05±10.036	61.30±9.943
LVEF (%)	47.11±12.36	43±11.715	48.078±12.327
Surgery duration (min)	269.46±58.50	276.87±61.083	267.04±57.540
LOH (day)	6.11±6.31	9.41±9.477	5.36±5.068
BMI (kg/m^2^)	27.43±43.43	27.57±4.56	27.40±4.40

Incidence of AKI and prevalence of risk factors

**Figure 1:**
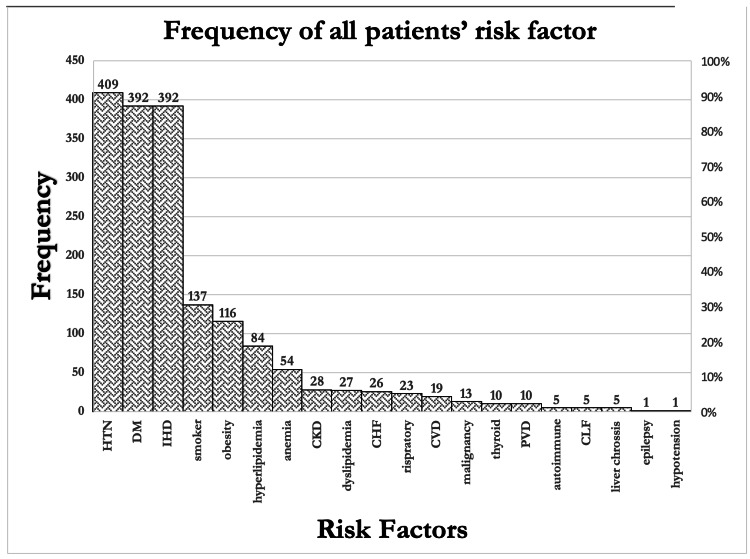
Frequency of Risk Factors among All the Patients Included in the Study HTN: hypertension; DM: diabetes mellitus; IHD: ischemic heart disease; CKD: chronic kidney disease; CHF: chronic heart failure; CVD: cardiovascular disease; PVD: peripheral vascular disease

Within the sample of 564 patients across all the years, 110 experienced an AKI event with an overall AKI rate of (19.5%) (Table 1). Comorbidities played an important role in the development AKIs after cardiac interventions. HTN was the most common comorbidity, 409 (72.5%). The second most common comorbidity was diabetes and IHD, both being present in 392 (69.5%) patients. Other risk factors included obesity, 116 (20.6%); smoking, 137 (24.3%); CKDs, 28 (5%); anemia, 54 (9.6%); and hyperlipidemias, 84 (14.9%) (Figure 1).

Multivariate analysis of preoperative risk factors associated with AKI

With regard to the comorbidities, we conducted binary logistic analyses and calculated the P-values, RRs, and CIs of all the variables, and the results were significant for some variables. Patients with diabetes were more likely to develop AKIs (P = 0.012, RR = 2.280, CI = 1.198-4.339). Furthermore, HTN had a strong effect on the occurrence of AKIs (P = 0.004, RR = 2.865, CI = 1.391-5.900). Moreover, patients who suffered from CHF were susceptible to AKIs (P = 0.008, RR = 4.189, CI = 0.512-1.493). In addition, anemia showed a significant P-value (0.002), and these patients were more likely to progress to AKIs (3.2 times) with CIs between 1.509 and 6.822 (Table 2). Patients with CKDs showed a greater progression to AKIs (P = 0.016, RR = 3.373, CI = 1.250-9.098). On the other hand, we compared males and females to determine whether there were any differences between the genders, and we found that males were less likely (P = 0.008, RR = 0.440, CI = 0.240-0.807) to develop AKIs compared with females (P = 0.008, RR = 2.005, CI = 1.135-3.542). The LVEF was also found to have a relationship with the development of AKIs (P < 0.005, RR = 1.041, CI = 1.018-1.064) (Table 2). Between the types of cardiac surgery, CABG was approximately five times more likely to lead to AKIs (P < 0.005, RR= 4.909, CI = 2.271-10.612) compared with PTCA (P < 0.05, RR = 0.204, CI = 0.094-0440). Furthermore, elective surgeries were less likely to lead to AKIs compared with emergency surgeries which have a significant risk for the development of AKIs (P < 0.002, RR = 2.687, CI = 1.431-5.046) (Table 2). Nonetheless, IHD did not show a significant development of AKIs (P > 0.632, RR = 0.875, CI = 0.512-1.49). Moreover, there were no associations between AKIs and cerebrovascular diseases (P > 0.381, RR = 1.812, CI = 0.479-6.856). Collectively, our data demonstrated that AKIs were not common in obese patients (P > 0.616, RR = 0.850, CI = 0.451-1.604) and in hyperlipidemic patients (P > 0.881, RR = 0.949, CI = 0.480-1.878) (Table 2).

**Table 2:** Multivariate Analysis of Preoperative Risk Factors Associated with AKI RR: relative risk; CI: confidence intervals; CLD: chronic liver disease; PVD: peripheral vascular disease; CKD: chronic kidney disease; CABG: coronary artery bypass graft surgery; PTCA: percutaneous transluminal coronary angioplasty; LVEF: left ventricular ejection fraction; LOH: length of hospitalization; BMI: body mass index. All Variables were analyzed by binary logistic regression test. Age, LVEF, LOH, BMI, and surgery duration were described by mean and SD, others by number and percentage.

Variable	Total number (N = 564)	Develop AKI (N = 110)	Non-develop AKI (N = 454)	P-value	RR	95% CI (lower-upper)
Gender(1): male	481 (85.3%)	81 (73.6%)	400 (88.1%)	<0.008	0.440	(0.240-0.807)
Gender(2): Female	83 (14.7%)	29 (26.4%)	54 (11.9%)	<0.008	2.005	(1.135-3.542)
Nationality(1): Saudi	87 (15.5%)	21 (19.4%)	66 (14.6%)	<0.242	1.462	(0.773-2.766)
Nationality(2): Non-Saudi	473 (84.5%)	87 (80.6%)	386 (85.4%)	<0.242	0.684	( 0.362-1.293 )
Risk Factors	Total number (N = 564)	Develop AKI (N = 110)	Non-develop AKI (N = 454)	P-value	RR	95% CI (Lower-Upper)
Diabetes mellitus	392 (69.5%)	94 (85.5%)	298 (65.5%)	<0.012	2.280	(1.198-4.339 )
Hypertension	409 (72.5%)	98 (89.1%)	311 (68.5%)	<0.004	2.865	(1.391-5.900)
Ischemic heart disease	392 (69.5%)	78 (70.9%)	314 (69.2%)	<0.623	0.875	(0.512-1.493)
Chronic heart failure	26 (4.6%)	11 (10%)	15 (3.3%)	<0.008	4.189	(1.452-12.087)
Malignancy	13 (2.3%)	0 (0%)	13(2.9%)	<0.999	0.000	
Cerebrovascular disease	19 (3.4%)	5 (4.5%)	14 (3.1%)	<0.381	1.812	(0.479-6.856)
Autoimmune disease	5 (0.9%)	0 (0%)	5 (1.1%)	<0.999	0.000	
Thyroid disease	10 (1.8%)	1 (0.9%)	9 (2%)	<0.555	0.458	(0.034-6.106)
CLD	5 (0.9%)	0 (0%)	5 (1.1%)	<1.000	0.573	
PVD	10 (1.8%)	1 (0.9%)	9 (2%)	<0.279	0.276	(0.027-2.842)
Respiratory disease	23 (4.1%)	3 (2.7%)	20(4.4%)	<0.258	0.435	(0.103-1.839)
CKD	28 (5%)	13 (11.8%)	15 (3.3%)	<0.016	3.373	(1.250-9.098)
Anemia	54 (9.6%)	18 (16.4%)	36 (7.9%)	<0.002	3.209	(1.509-6.822)
Hyperlipidemia	84 (14.9%)	17 (15.5%)	67 (14.8%)	<0.881	0.949	(0.480-1.878)
Obesity	116 (20.6%)	22 (20%)	94 (20.7%)	<0.616	0.850	(0.451-1.604)
Liver cirrhosis	5 (0.9%)	0 (0%)	5 (1.1%)	<0.999	0.000	
type of surgery(1): CABG	407 (72.2%)	100 (90.9%)	307 (67.6%)	<0.000	4.909	(2.271-10.612)
type of surgery(2): PTCA	157 (27.8%)	10 (9.1%)	149 (32.4)	<0.000	0.204	(0.094-0.440)
priority of surgery(1): Elective	492 (87.2%)	81 (73.6%)	411 (90.5%)	<0.002	0.372	(0.198-0.699)
priority of surgery(2): Emergency	72 (12.8%)	29 (26.4%)	43 (9.5%)	<0.002	2.687	(1.431-5.046)
Age (years)	61.64±9.97	63.05±10.036	61.30±9.943	<0.632	0.0994	(0.968-1.020)
LVEF (%)	47.11±12.36	43±11.715	48.078±12.327	<0.000	1.041	(1.018-1.064)
LOH (Days)	6.11±6.31	9.41±9.477	5.36±5.068	<0.005	0.943	(0.905-0.982)
BMI (kg/m^2^)	27.43±43.43	27.57±4.56	27.40±4.40	<0.475	0.970	(0.0891-1.055)
Surgery duration (min)	269.46±58.50	276.87±61.083	267.04±57.540	<0.109	0.996	(0.992-1.001)

Laboratory tests

Paired-sample t-tests were conducted to assess the changes in the creatinine levels. A statistically significant increase in creatinine levels was observed from time 1; preoperative (mean and SD = 96.47±44.98) to time 2; postoperative (mean and SD = 143.67±105.40, t (520) = 12.595, P <0.000). On the other hand, using binary logistic analyses, we found no relationships between the preoperative hemoglobin (P = 0.779), BUN (P = 0.648), HCT (P = 0.979), and the development of AKIs, as shown in Table 3.

**Table 3:** Multivariate Analysis of Pre- and Postoperative Laboratory Results Associated with AKI Data presented as: mean ± standard deviation; P-value; RR: relative risk; CI: confidence intervals. Hb: hemoglobin; BUN: blood urea nitrogen; HCT: hematocrit

Variable	Total Number (N = 564)	Develop AKI (N = 110)	Non-develop AKI (N = 454)	P-value	RR	95% CI (Lower-Upper)
Preoperative creatinine	96.47±44.98	119.399±74.45	90.89±31.91	<0.000	1.183	(1.116-1.255)
Postoperative creatinine	143.67±105.40	277.92±153.51	107.92±41.84	<0.000	0.860	(0.820-0.902)
Preoperative Hb	13.09±2.05	12.28±2.26	13.29±1.95	<0.779	0.827	(0.219-3.121)
Preoperative BUN	5.97±3.29	7.65±4.46	5.57±2.81	<0.648	0.932	(0.690-1.260)
Preoperative HCT	39.28±5.69	37.33±6.92	39.74±5.46	<0.932	0.979	(0.605-1.584)

## Discussion

Prevalence of AKIs

One of the most important findings in our study was that the prevalence of AKIs after cardiac interventions was 19.5%. Studies that were conducted in other countries such as China [24], Malaysia [30], Belgium [25], and Canada [31] showed higher percentages of developing AKIs after cardiac interventions, than the percentage identified in our study. This difference may have been to factors such as better diagnostic tools, the means of the ages since older ages are more prone to developing AKIs, and better documentation of the patient’s profiles.

Hypertension and AKI

In our study, HTN was the most common comorbidity that the patient had before the intervention. The RR of HT was found to be very significant in our study and similar to two other studies that were conducted in Shanghai Renji Hospital, China [24] and the University of Florida, USA [32]. However, on the other hand, another study that was conducted in Malaysia showed that the RR of HTN was lower than that in our study [30]. These differences in the results may be due to that HTN being common in our country, our poor lifestyle habits, and low awareness of the importance of screening in our population.

Diabetes mellitus and AKI

In our study, diabetes was the second most common comorbidity that the patients presented with significant P-values and results for its RR. While other studies that were conducted in China [24], the USA [33], and Malaysia [30] also showed significant results, they were not as high as those in our study. This may be explained by the differences in the occurrence of diabetes between countries. Saudi Arabia is one of the countries most affected by diabetes may be due to the poor control of diabetes in our population. Overall, diabetes affects the small vessels in the kidney, which become narrow and clogged. Without adequate blood flow, the kidney becomes damaged, leading to functional impairment.

Anemia and AKI

Anemia is one of the major comorbidities that contributed to the development of AKIs following the interventions. Additionally, studies conducted in the USA [34], Malaysia [30], and Canada [35], with approximately the same results, and with all showing a high percentage of developing AKIs, concluded that anemia is a very dangerous risk factor. Anemia is a major comorbidity mainly because it decreases tissue oxygen delivery leading to renal medullary hypoxia and the development of AKIs. Additionally, intraoperative transfusions have been found to increase the risk of postoperative AKI development.

Chronic heart failure and AKI

Patients with CHF are in greater danger of AKIs. A previous study conducted at the University Hospital Vienna, Austria [10] and at the University of Florida, USA [32] found a similar significant relationship between AKIs and CHFs, which increased the incidence of AKIs and the rate of mortality in patients who underwent cardiac surgeries with CHFs as preoperative comorbidities. It is considered that CHF changes the normal physiological function of the kidney and with the addition of greater stress like undergoing surgeries, the kidneys become vulnerable to damage.

CABG surgeries and AKI

CABG surgery was the most common intervention that was performed in our study with the findings suggesting a relationship between CABG surgeries and AKIs. Furthermore, a study that was conducted in China showed a higher likelihood of the development of AKIs after CABG surgeries [24]. Additionally, a study that was conducted in the USA [33], also showed a significant association between them, and a further study that was also conducted in the USA [32] showed the same results with a positive association between the two factors. These findings were all significant which may have been due to CABG surgery being an invasive procedure, prone to many complications, and also because the patients who underwent this type of surgery had serious comorbidities.

Age and AKI

In this study, the mean age of the patients who developed AKIs was 63.05±10.03. Studies conducted in Sweden [34] and the USA [36] showed approximately the same mean for the ages of patients that developed AKIs after the interventions. These mean ages may have been due to the comorbidities that affected old ages making them more prone to the development of AKIs.

Priority of surgery and AKI

From the SPSS data analysis, emergency surgeries showed a significant correlation with elective surgeries. The Cleveland Clinic Foundation identified urgent surgery as an important factor in the development of AKIs [6]. We considered the development of AKIs to be a logical result of patients who came to the hospital in bad condition.

AKI and surgery duration

With respect to the surgery duration, this study did not show a relationship with AKIs. Furthermore, a study that was conducted in the USA [37] did not show a significant relationship between AKIs and surgery durations, whereas a study that was conducted in Asia [30] showed a strong association between the two factors. This difference between the studies may be due to complications that can occur during the procedure which increase the surgery duration and which may lead to the development of AKIs.

Limitations

This study had a number of limitations, which should be taken into account when interpreting the findings. First, the study was retrospective in nature and was conducted at a single organization. This made the findings of the study prone to bias and it also limited its generalizability. Second, since the Phoenix system was established in 2013 at KAUH, we faced difficulties in obtaining patients’ information and laboratory tests from the period between 2010 and 2013. Third, we did not have an accurate measurement of the preoperative glomerular filtration rate, which indicated that some patients with preexisting mild renal disease may have been included. Fourth, the type of acute renal failures (ischemic, nephrotoxic, or atheroembolic), as well as the stages of the AKI, could not be determined precisely due to poor documentation. Finally, due to the lack of urine output values, only the creatinine values were used to determine whether or not a patient met the criteria for AKIs.

## Conclusions

In summary, this study explored the relationship between cardiac interventions and AKIs in adults, between 2010 and 2020, at KAUH in Jeddah, Saudi Arabia. Acute kidney injuries are common complications in adults after cardiac interventions, especially after CABG surgeries, and they are linked to poor outcomes. According to our findings, DM, HTN, CHF, CKD, and anemia are risk factors associated significantly with the development AKIs in patients. Other studies have also indicated that patients who underwent cardiac surgeries with these risk factors had the potential to develop AKIs compared with healthy individuals. Moreover, we need to identify these risk factors and manage them appropriately to avoid the complication of cardiac interventions. Furthermore, we recommend being more aware of these comorbidities and not ignore them, to better understand the burden of kidney injuries.
